# ^Contribution of fortified margarines and other plant-based fats to micronutrient intake in the Netherlands^

**DOI:** 10.1007/s00394-021-02757-z

**Published:** 2022-01-01

**Authors:** Marjolein H. de Jong, Eline L. Nawijn, Janneke Verkaik-Kloosterman

**Affiliations:** National Institute for Public Health and the Environment (RIVM), Bilthoven, The Netherlands

**Keywords:** Food fortification, Micronutrients, Margarines, Plant-based fats, The Netherlands, Habitual intakes

## Abstract

**Purpose:**

In the Netherlands, margarines and other plant-based fats (fortified fats) are encouraged to be fortified with vitamin A and D, by a covenant between the Ministry of Health and food manufacturers. Frequently, these types of fats are also voluntarily fortified with other micronutrients. The current study investigated the contribution of both encouraged as well as voluntary fortification of fortified fats on the micronutrient intakes in the Netherlands.

**Methods:**

Data of the Dutch National Food Consumption Survey (2012–2016; *N* = 4, 314; 1–79 year.) and the Dutch Food Composition Database (NEVO version 2016) were used to estimate micronutrient intakes. Statistical Program to Assess Dietary Exposure (SPADE) was used to calculate habitual intakes and compared to dietary reference values, separate for users and non-users of fortified fats.

**Results:**

Of the Dutch population, 84% could be considered as user of fortified fats. Users consumed mostly 1 fortified fat a day, and these fats contributed especially to the total micronutrient intake of the encouraged fortified micronutrients (vitamins D and A; 44% and 29%, respectively). The voluntary fortification also contributed to total micronutrient intakes: between 7 and 32%. Vitamin D and A intakes were up to almost double among users compared to non-users. Intakes were higher among users for almost all micronutrients voluntarily added to fats. Higher habitual intakes resulted into higher risks of excessive vitamin A-intakes among boys and adult women users.

**Conclusion:**

Consumption of fortified fats in the Netherlands resulted into higher vitamin A and D-intakes among users, compared to non-users of these products.

## Introduction

To support health, optimal micronutrient (vitamins and minerals) intakes are essential [1]. Since inadequate micronutrient intakes may lead to deficiency diseases, sufficient micronutrient intake should be promoted. On the contrary, excessive intakes should be avoided, since these might result into adverse health effects. In many countries, including the Netherlands, at least for some micronutrients the intakes are suboptimal [2].

Food fortification is a common strategy to improve micronutrient intakes in a whole population. In the Netherlands, however, low micronutrient intake is generally a problem in specific subgroups, therefore often a subgroup-specific supplementation advice is given [3]. With food fortification, micronutrients may be added voluntarily to foods, yet enrichment can also be mandated by governments. In the European legislation requirements regarding food fortification are foreseen. However, maximum fortification levels have not been set yet. Consequently, in several European Member States there is national legislation for food fortification [4].

In the Netherlands, mandatory fortification is legally not feasible [5]. But, voluntary food fortification is allowed for most of the micronutrients. In addition, to encourage the addition of specific micronutrients to specific food products to increase the population’s intake, covenants between (Dutch) food manufacturers, supermarket trade organisation (food service companies) and the Dutch government are set. In these covenants it is agreed to add specific micronutrients to specific food groups and also the amounts added are specified.

Since 1999, there is a covenant for the fortification of margarines and other spreadable fats with vitamins A and D [5, 6]. Prior to 1999, addition of vitamins A and D to margarines and other spreadable fats was mandatory. However, due to a court decision [7], mandatory fortification is not feasible anymore. Therefore the Dutch legislation changed. With the covenant virtually all these fats contain added vitamins A and D. Exception may be a few products not manufactured in the Netherlands, and as such not per se falling under the covenant. In this covenant a vitamin A-content between 6 and 8 µg per gram product (retinoid form) and a vitamin D-content between 0.056 and 0.075 µg per gram product was agreed. Fortification of margarines with vitamin A and/or D is also encouraged in other countries, for example in Finland, Belgium, Poland and Sweden [8]. There is, however, little known on the contribution of the (semi-) mandatory fortification of margarines on the vitamin A and D-intakes. Jääskeläïnen et al*.* (2017) showed this (semi-) mandatory fortification had a positive impact on the vitamin D status of the Finnish population, but how it affects intakes in other population is not known.

Besides the encouraged fortification with retinol and vitamin D, it is also possible to voluntarily add micronutrients to foods, including margarines and other plant-based fats [5]. Consequently, some fats also contain other added micronutrients like folic acid or vitamin E.

Within the Dutch population, low vitamin A intakes are observed among adolescents and adults and no statement can be made on the risk on D-deficiency [9]. Besides that, low calcium, vitamin B2, C and folate-intakes are observed for Dutch adults and median calcium, vitamin B1, B2 and folate-intakes below the adequate intake were observed among children, which are all nutrients voluntarily added to margarines. What the effect of voluntary fortification of fats fortified with vitamin A and D, is not known. Therefore, the aim of this study is to investigate the contribution of both the encouraged as well as the voluntary fortification of margarines and other plant-based fats on the micronutrient intake in the Netherlands and to evaluate the intake by comparing with dietary reference values.

## Methods

### Survey population

In the Dutch National Food Consumption Survey (DNFCS) 2012–2016 data were collected on food consumption of the Dutch population (*N* = 4313, 1–79 yr.). A detailed description of the DNFCS 2012–2016 is described elsewhere [9]. Briefly, a sample representative for the Dutch population was selected from a consumer’s panel (Kantar TNS; net response rate 65%). Pregnant and lactating women were excluded from participation, as well as institutionalized people. Only people with adequate command of the Dutch language were included.

Food consumption data were collected with two non-consecutive 24-h recalls, with the interview programme GloboDiet (IARC^©^; formerly EPIC-Soft). All interviews were performed by trained dieticians. Children 4–15 years old were interviewed at home together with a parent or caretaker. For younger children, the parents or caretakers were interviewed about their child’s food consumption. Participants aged 16 to 70 years old were interviewed by phone without prior notification. For the youngest (1–8 years) and oldest (71–79 years) age groups the 24-h recalls were combined with food diaries and these interviews were performed at home. To be able to estimate the micronutrient intake, the food consumption data was linked to the Dutch Food Composition Database (NEVO; NEVO-online version 2016/5.0, RIVM, Bilthoven, 2016, with additions for the DNFCS 2012–2016).

The age of the respondent was defined as the age at the first 24-h recall day. Height and weight were measured on the first recall day for children up to 15 years and older adults 71–79 years old and self-reported for participants 16–70 years old. Body mass index (BMI) was calculated per person as the body weight divided by the height squared (kg/m^2^) and categorized for adults into seriously underweight and underweight (BMI < 18.5), normal weight (BMI between 18.5 and 25) and overweight and obesity (BMI > 25). For children the same categories were used, but cut-off points for children were lower than those for adults and differ among months of age [10]. Other general characteristics of the participants were collected by a general questionnaire. The questions covered various background factors (such as educational level, working status, native country, family size), various life style factors (such as patterns of physical activity, smoking and use of alcoholic beverages) and various general characteristics of the diet (such as special diets and eating habits). The questionnaires differed between age groups, to take into account differences in the way of living. The degree of urbanisation was divided in extremely urbanised (2500 or more addresses/km^2^), strongly (1500–2500 addresses/km^2^), moderately (1000–1500 addresses/km^2^), hardly (500–1000 addresses/km^2^) and not urbanised (fewer than 500 addresses/km^2^). The educational level concerned the highest completed educational level of the participants or, in case of participants ≤ 18, of the head of household, categorised into low (primary education, lower vocational education, advanced elementary education), middle (intermediate vocational education, higher secondary education) and high (higher vocational education and university).

### Definition fortified margarines and other plant-based fats (fortified fats)

Margarines and other plant-based fats fortified with retinol and vitamin D were considered as fortified fats in this study and selected based on the ingredient declaration of the fats. Users were defined as those subjects consuming at least one fortified fat on at least one of the recall days. Non-users were identified as those who did not consume any fortified fat on both recall days. In this study, we only considered the micronutrient intake from foods and drinks, intake from dietary supplements was excluded.

### Variation in- and amount of fortified fats consumed

Within NEVO, all details of same types of foods with comparable compositions are merged within one NEVO-code, sometimes this codes includes several brands with more or less equal composition [11]. To study how many different fortified fats users had consumed on a recall day, fortified fats were differentiated by their NEVO-code. Therefore, fortified fats with the same NEVO-code were considered as one type of fat, and only counted once.

It is also possible that users consumed the same fortified fat during multiple mealtimes a day. A mealtime was defined as each moment a participant ate a meal (breakfast, lunch, dinner) or a snack (before breakfast, between breakfast and lunch, between lunch and dinner, after dinner) on a recall day. To investigate how often fortified fats were consumed on a recall day, the amount of consumed fortified fats by each user on a recall day was calculated. Within the same mealtime moment, the consumption of multiple portions of the same fortified fat (based on the same NEVO-code) was only counted once. If, however, the same fortified fat was consumed within for example two different mealtimes (e.g. breakfast and lunch), it was counted twice.

### Contribution of fortified fats to total micronutrient intake

To study the contribution of fat fortification, the contribution of micronutrient intakes from these fats to total micronutrient intake from all consumed foods and drinks per recall day was calculated. This was only calculated for users of the fats fortified with that specific micronutrient for the micronutrients added to fortified fats: calcium, vitamin A (retinol as well as retinol activity equivalents (RAE)), vitamins B_1_, B_2_, B_3_, B_6_, B_12_, D and E, and folate equivalents (both folate naturally present in foods as well as synthetic folic acid). For vitamin A expressed as RAE, 1 μg RAE was assumed to be equal to 1 μg retinol, 12 μg β-carotene and 24 μg other carotenoids [12]. Folate equivalents were calculated as the amount of folate naturally present in foods (in μg) and 1.7 times the amount of folic acid in enriched foods (in μg) [13]. Vitamin K was not included in this study as the coverage of this micronutrient in the NEVO-database was too low (49%). In addition, vitamin K is not added to fats in the Netherlands. All users of fortified fats have a contribution of these fats to their total retinol and vitamin D intake, due to the encouraged fortification. For all other voluntarily added micronutrients the contribution from fortified fats was only calculated for the users of the fats fortified with that specific micronutrient.

### Habitual micronutrient intakes

The habitual micronutrient intake (also referred to as usual intake) distribution of both non-users and users of fats fortified with that specific micronutrient were estimated by correcting the data for the within-person variation using the Statistical Program to Assess Dietary Exposure (SPADE; version 4.0.85 of 16 December 2020) and compared to each other [14]. With bootstrap (200 iterations), the 95% confidence intervals (95% CI) were calculated. Intakes were modelled as a function of age, using the SPADE 1-part model. From this, the habitual intake distribution was presented into four age-sex classes; boys 1–17 year old, girls 1–17 year old, men 18–79 year old and women 18–79 year old. These analyses were performed in R version 1.1.383.

### Assessment of risk on inadequate and/or excessive micronutrient intakes

To assess the risk on inadequate and/or excessive micronutrient intakes, dietary reference values established by the Health Council of the Netherlands were used [12, 15]. For vitamin D, habitual intakes were compared to both the reference values established for assumed insufficient and sufficient sunlight exposure. For vitamin A and E, an adequate intake (AI) was established for children aged 1–13 and an estimated average requirement (EAR) for children aged 14–17 years old. For all other nutrients, an AI was established for all children.

For micronutrients with an EAR, the proportion of the population with inadequate habitual intakes was estimated with the EAR cut-point method [16]. The adequacy was qualitatively assessed for micronutrients with an AI. A habitual median intake above the AI was considered as a low risk of inadequate micronutrient intake [17]. If the median habitual intake was below the adequate intake, no statement about the adequacy could be made. Also, the proportion of the population with a habitual micronutrient intake above the upper level (UL) was estimated for those nutrients for which an upper level was established: vitamin A (retinol), D, B6, E and calcium.

For vitamin B1 and B3 the number of users was small (*N* = 102 and 46, respectively). Therefore, it was not possible to calculate the 95% CI and to assess if intakes were significantly different between users and non-users or calculate the proportions above the UL or below the EAR.

### Statistical analysis

Due to the study design of the DNFCS 2012–2016, children were overrepresented in the sample to obtain equal age groups [9]. To make the results representative for the Dutch population (calendar year 2014), a weighting factor was applied in the analyses. This weighting factor included socio-demographic factors, as well as season and day of the week. In general, the deviations of the weighted results from the unweighted were small, therefore only the weighted results were presented.

General characteristics of users and non-users of fortified fats were compared with a Chi-Square test. A *p* value < 0.05 was considered to indicate a statistically significant difference between the groups. *P* values were corrected for multiple comparisons using the Bonferroni correction [18].

The contribution of micronutrient intake from fortified fats to total micronutrient intake was calculated for each recall day a fortified fat was consumed, meaning no average intake over two recall days, or an habitual intake were calculated, as some users only had micronutrient intake from fortified fats on one recall day. For these users, the other recall day was not included in the analysis for contribution to total micronutrient intake.

To compare habitual intakes and proportions below the EAR and above the UL of users and non-users, the difference was calculated with the accompanying 95% CI (bootstrap 200 iterations), similar to Dekkers and Slob [19]. 95% CI of the differences not including zero were considered as statistically different between users and non-users.

Unless otherwise stated, statistical analyses were performed using SAS version 9 (SAS Institute Inc., Cary, NC; Windows version 6.3.9600).

## Results

### Characteristics of the study population

A large part of the Dutch population was a user of fortified fats on at least one of the two recall days (Table 1). Among the users of fortified fats there were significantly more people with a lower, or middle education, people living in moderately, hardly or not urbanized areas, and people with a Dutch ethnicity, compared to the non-users.

**Table 1:** Weighted characteristics of users and non-users of fortified fats ^a,b^

	Non-users (*N* = 687)	Users (*N* = 3626)	Adjusted *p* value^c^
Sex			1.000
Men	347 (50%)	1818 (50%)	
Women	340 (50%)	1808 (50%)	
Age			0.096
Children (1–17 years)	115 (17%)	751 (21%)	
Adults (18–79 years)	571 (83%)	2875 (79%)	
BMI^d^			1.000
(extremely) Underweight	21 (3%)	107 (3%)	
Normal weight	335 (51%)	1619 (48%)	
Overweight/obese	301 (46%)	1632 (49%)	
Smoking^e^			1.000
Yes	105 (19%)	667 (24%)	
No	461 (81%)	2171 (76%)	
Alcohol user^e^			1.000
Yes	419 (73%)	2114 (74%)	
No	153 (27%)	761 (26%)	
Following a diet			0.159
Yes	122 (18%)	458 (13%)	
No	564 (82%)	3168 (87%)	
Sports			0.233
Yes	318 (52%)	1366 (48%)	
No	248 (44%)	1472 (52%)	
Days a week 1 h activity			1.000
3 or less	15 (23%)	80 (27%)	
4 or 5	10 (15%)	41 (14%)	
6 or 7	40 (62%)	171 (59%)	
Educational level ^f^			** < .0001**
Low	101 (15%)	952 (26%)	
Middle	278 (40%)	1562 (43%)	
High	308 (45%)	1112 (31%)	
Urbanisation ^g^			**0.007**
Extremely/strongly	391 (57%)	1669 (46%)	
Moderately	115 (17%)	742 (20%)	
Hardly/not	180 (26%)	1215 (33%)	
Migration background			**0.018**
Dutch	609 (89%)	3360 (93%)	
Western immigrant	14 (2%)	97 (3%)	
Non-Western immigrant	62 (9%)	169 (5%)	
Season (First recall day)			1.000
Spring	188 (27%)	891 (25%)	
Summer	157 (23%)	921 (25%)	
Autumn	186 (27%)	892 (25%)	
Winter	155 (23%)	923 (25%)	
Recall days			1.000
Weekend/week	323 (47%)	1790 (49%)	
Only week	223 (33%)	1200 (33%)	
Only weekend	140 (20%)	636 (18%)	

^a^Weighted for socio-demographic factors, season and day of the week

^b^Not all characteristics were collected for all participants and as a result of the use of a weight factor, results needed to be rounded to numbers without decimals, resulting into some groups of non-users and/or users with an n not equal to 687 and/or 3626

^c^*P* value calculated with the Chi-square test, and corrected with the Bonferroni correction

^d^For children age specific cut-off values were used. For adults: (extremely) Underweight: BMI < 18.5, normal weight BMI = 18.5–25, overweight/obesity: BMI > 25

^e^For participants ≥ 18 years old

^f^The highest education of the parents for children

^g^Extremely/strongly urbanised: > 1500 addresses/km^2^, moderately urbanised: 1000–1500 addresses/km^2^, hardly/not urbanised: < 1000 addresses/km^2^

### Consumption of fortified fats

Most users (76%) consumed fortified fats on both recall days. This means that 24% of the users consumed fortified fats on 1 recall day. On more than half of the recall days only 1 type of fortified fat was consumed, which was mostly a spreadable fat (74%). On 29% of the recall days two types of fortified fats were consumed, this was mostly (88%) a spreadable fat and a fat used for cooking.

Some users also used the same fortified fat (based on the same NEVO-code) during multiple mealtimes a day. Counting the same fortified fat multiple times if they were consumed on multiple mealtimes a day, mostly 1, 2, or 3 fortified fats were consumed throughout a recall day (28%, 28% and 18%, respectively).

### Contribution of fortified fats to total micronutrient intake

**Fig. 1:**
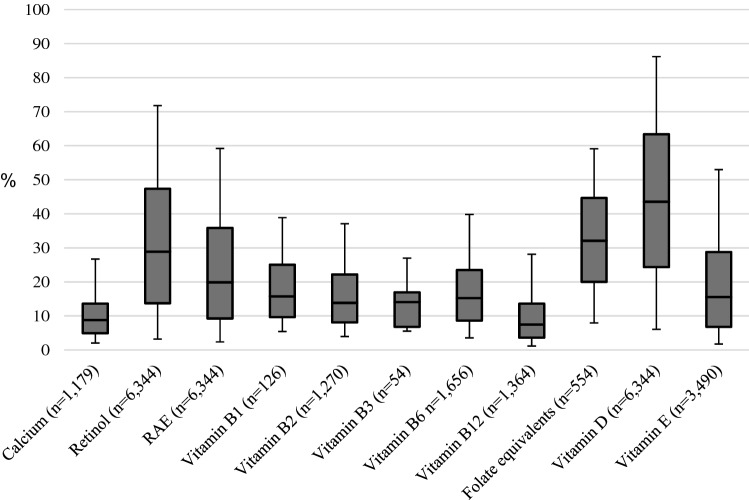
Contribution of fortified fat consumption to the total micronutrient intakes of users of fortified fats fortified with these specific nutrients. Lower whisk: P5, bottom boxplot: P25, line in middle: P50 (median), top boxplot: P75, upper whisk: P95, n: number of recall days on which a fat fortified with that specific nutrient was consumed

Among all fortified fat users, the median contribution of fortified fats to the total vitamin D and A (retinol and RAE) intake was 44%, 29% (retinol) and 20% (RAE), respectively (Fig. 1). Besides vitamins A and D, some of the consumed fats were also fortified with calcium, vitamin B1, B2, B3, B6, B12, folic acid and vitamin E. The median contribution of fats fortified with these specific nutrients varied between 7% for vitamin B12 to 32% for folic acid (expressed as folate equivalents). The number of users of voluntarily fortified fats were however relatively small compared to the group using fortified fats with vitamin D and vitamin A. For example fats fortified with vitamin B3 were only consumed on 1% of all recall days and folic acid on 6% of the recall days. For vitamin B6 and vitamin E the number of users was higher and were consumed on respectively 19% and 40% of all recall days. Among users there is variation in the contribution of fortified fats to the micronutrient intake. Variation (P5-P95) is large for retinol (3–72%) and vitamin D (6–86%).

### Habitual intakes

**Fig. 2:**
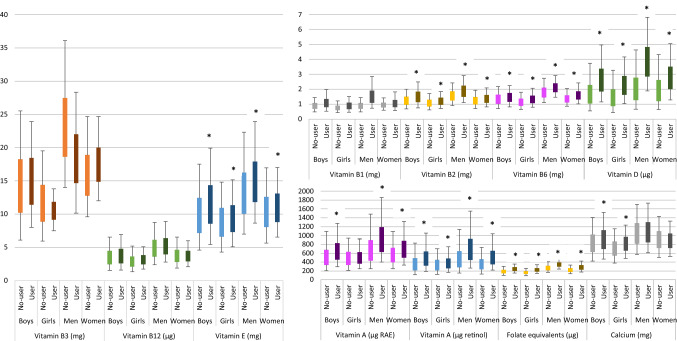
Habitual intake distribution for users and non-users. Lower whisk: P5, bottom boxplot: P25, line in middle: P50, top boxplot: P75, upper whisk: P95, *significant higher median habitual intake among users (not possible for vitamin B1 and B3)

Users of the encouraged fortified fats had median habitual intakes up to 40% higher for vitamin A and almost twice as high vitamin D intakes, compared to non-users (Fig. 2). For vitamin D this difference between users and non-users of fortified fats was statistically significant for all studied age-gender groups. For vitamin A this was also the case for boys and adults. For girls the vitamin A intake was not statistically significantly different between users and non-users of fortified fats.

The higher vitamin A-intakes lead to a lower proportion of intakes below the EAR among users, compared to non-users (49–64% among non-users and 23–43% among users; Table 2), except for the girls.

**Table 2:** The assessment of inadequacy of micronutrient intakes separate for users and non-users of fortified fats

Micronutrient	Age (year)	Gender	AI	EAR	Non-users		Users	
					Evaluation risk inadequate intake for each age (year)	% < EAR	Evaluation risk inadequate intake for each age (year)	% < EAR^a^
Vitamin A (µg RAE)	1–17	Boys	300/350/400/ 600^b^	600^c^	1–9: LR 10–13: NSP	64.4 (55.6–69.3)	1–9, 11–13: LR 10: NSP	**42.6 (37.6–47.7)**
		Girls		500^c^	1–9: LR 10–13: NSP	53.1 (48.5–61.5)	1–9: LR 10–13: NSP	48.7 (44.0–54.3)
	18–79	Men	–	615	–	49.4 (43.1–54.1)	–	**23.3 (19.6–27.0)**
		Women	–	525	–	47.6 (42.5–52.7)	–	**29.2 (25.7–33.5)**
Vitamin D (µg)	1–17	Boys	10/3^d^	–	NSP/ NSP^5^	–	NSP/ 1: LR 2–17: NSP^5^	–
		Girls			NSP/ NSP^5^	–	NSP/ NSP^5^	–
	18–79	Men	10/3^d^	10^g^	NSP/ NSP^d^	99.9 (99.5–100.0)	NSP/ LR^d^	99.6 (99.0–100.0)
		Women			NSP/ NSP^d^	99.8 (99.6–100.0)	NSP/ NSP^d^	99.9 (99.9–100.0)
Vitamin B1 (mg)	1–17	Boys	0.3/0.5/0.8/1.1	–	1–13: LR 14–17: NSP	–	1–13, 16–17: LR 14–15: NSP	–
		Girls			1–8: LR 9–17: NSP	–	1–13: LR 14–17: NSP	–
	18–79	Men	–	0.072	–	–	–	–
		Women	–		–	–	–	–
Vitamin B2 (mg)	1–17	Boys	0.5/0.7/1/1.5	–	1–13: LR 14–17: NSP	–	1–13: LR 14–17: NSP	–
		Girls	0.5/0.7/1/1.1		1–9, 11: LR 10, 12–17: NSP	–	1–13, 15: LR 14, 16–17: NSP	–
	18–79	Men	–	1.3	–	29.9 (27.1–35.1)	–	**13.4 (9.8–18.3)**
		Women	–		–	59.4 (56.0–62.7)	–	**45.6 (40.7–51.2)**
Vitamin B3 (mg)	1–17	Boys	4/7/11/17	–	1–13, 15–17: LR 14: NSP	–	1–13, 15– 17: LR 14: NSP	–
		Girls	4/7/11/13		1–13, 15–17: LR 14: NSP	–	1–13, 15–17: LR 14: NSP	–
	18–79	Men	–	1.3	–	–	–	–
		Women	–		–	–	–	–
Vitamin B6 (mg)	1–17	Boys	0.4/0.7/1.1/1.5	–	1–13: LR 14–17: NSP	–	1–13, 15–17: LR 14: NSP	–
		Girls			1–13: LR 14–17: NSP	–	1–13: LR 14–17: NSP	–
	18–79	Men	–	1.1	–	9.4 (7.5–12.7)	–	**0.7 (0.2–1.7)**
		Women	–		–	23.1 (19.4–28.1)	–	**9.5 (5.4–15.3)**
Folate equivalents (µg)	1–17	Boys	85/150/225/300	–	1–8: LR 9–17: NSP	–	1–8: LR 9–17: NSP	–
		Girls			1–8: LR 9–17: NSP	–	1–3, 5–8: LR 4, 9–17: NSP	–
	18–79	Men	–	200	–	16.3 (13.8–19.1)	–	**0.8 (0–1.8)**
		Women	–		–	38.6 (34.6–42.6)	–	**11.2 (6.8–15.0)**
Vitamin B12 (µg)	1–17	Boys	0.7/1.3/2/2.8	–	1–13, 15–17: LR 14: NSP	–	LR	–
		Girls			1–13, 17: LR 14–16: NSP	–	1–13, 15–17: LR 14: NSP	–
	18–79	Men	–	2	–	2.0 (1.1–2.9)	–	**0.4 (0–1.3)**
		Women	–		–	6.3 (4.9–9.3)	–	**3.9 (2.3–5.8)**
Vitamin E (mg)	1–17	Boys	4/5/6/8^b^	6^c^	LR	3.3 (1.2–5.3)	LR	**0.5 (0.1–1.0)**
		Girls	4/5/6/7^b^	5^c^	LR	3.9 (2.0–6.4)	LR	**1.3 (0.3–2.5)**
	18–79	Men	13	–	26–31, 33–41, 43–48, 51: LR 18–25, 32, 42, 49–50, 52–79: NSP	–	18–65: LR 66–79: NSP	–
		Women	11		70–73, 78–79: LR 18–69, 74–77: NSP	–	79: LR 18–78: NSP	–
Calcium (mg)	1–17	Boys	500/700/1200	–	1–8: LR 9–17: NSP	–	1–8: LR 9–17: NSP	–
		Girls	500/700/1100		1–3: LR 4–17: NSP	–	1–8: LR 9–17: NSP	–
	18–79	Men	1100/ 1200^e^	860/ 750^f^	NSP	37.8 (37.3–43.3)/16.6 (14.5–22.1) ^f^	NSP	31.8 (26.6–38.2)/14.9 (11.7–21.4) ^f^
		Women			NSP	61.1 (54.6–65.7)/29.6 (25.4–33.1) ^f^	NSP	58.4 (44.0–67.0)/32.3 (24.3–39.9) ^f^

*LR* low risk, *NSP* no statement possible

^a^Statistical significant lower proportion of users below the EAR compared to non–users is indicated when valued are displayed **bold**

^b^The AI accounts only for children 1–13 years old

^c^The EAR for children accounts only for children 14–17 years old

^d^Two AI-values for vitamin D, where 3 µg/day indicates adequate vitamin D intake with enough sun exposure and 10 µg/day if this amount of sun exposure is not met

^e^AI for women aged 50–79 and men 70–79 year old

^f^EAR = 860 for adults aged 18–24 year. EAR = 750 for women 25–49 year old and men 25–69 year old

^g^EAR only established for 70 years and older

Although the vitamin D intake is higher among users of fortified fats, the median intake remains below the AI (limited sun exposure 10 µg/day as well as sufficient sun exposure 3 µg/day) and no statement on the risk on inadequate vitamin D-intake could be made (Table 2). This is a similar conclusion as for non-users of fortified fats. Except for male users of fortified fats; their median vitamin D intake is higher than the AI with adequate sun exposure assumed, whereas this is not the case for non-users of fortified fats.

Among users of fats voluntarily fortified with other micronutrients than retinol and vitamin D, higher median habitual intakes were observed for calcium (children), folate-equivalents, vitamin B2, B6 and E (Fig. 2). Differences between users and non-users were smaller compared to the vitamin A and D intakes. The higher intakes resulted for these micronutrients in a higher proportion meeting the requirements (Table 2). For adult men, this resulted in a half less users down to almost no users with intakes below the EAR for vitamin B2 and folate equivalents. Although the median habitual intake was not higher among users of fortified fats compered to non-users, a higher proportion of users met the requirements for vitamin B12.

The higher intakes among users of fats encouraged and voluntarily fortified fats lead to significant higher proportions of boy and adult women-users of fortified fats above the UL for retinol compared to non-users. 3.7% of the boy users (95% CI 1.8–3.8) had intakes above the UL versus 1.5% of the non-users (95% CI 0.4–2.1). For adult women, this percentage was 0.3% among users (95% CI 0.2–0.9) versus 0% (95% CI 0–0.1). For the other age-sex groups for retinol and for everyone for the other micronutrients (vitamin D, B6, E and calcium), no significant difference was found and the percentage above the UL remained below 1% within both groups.

## Discussion

With 84% of the Dutch population being a consumer of fortified fats, fortified fat consumption is very common in the Netherlands. Most of these users consumed fortified fats on each recall day. The contribution of the fortified fats to the total intake of users was 29% and 44%, respectively. The consumption of fortified fats resulted in higher total vitamin A and D-intakes and consequently a lower proportion with inadequate vitamin A intake from food compared to non-users (except for girls), while no statement regarding adequacy was possible for vitamin D, except for adult male users taking the AI with assumed adequate sun exposure. In addition, fats voluntarily fortified with other micronutrients than vitamin A and D contributed between 7 and 32% to total intakes of micronutrients. For folate equivalents and vitamin B2, B6 and E, habitual intakes were higher among users, compared to non-users. However, the number of consumers of fats fortified with these micronutrients was low.

Our study shows that the largest effect on intake is observed for vitamin A and D. For both these nutrients fortification of fats is encouraged via a covenant between the food producers and the ministry of Health, Welfare and Sport. Consequently, all these fats contain at least 6 µg vitamin A (retinoid form) and 0.056 µg vitamin D per gram product. Fats fortified with other nutrients is less common and the effects on intake at population level are therefore smaller, although the effects could be substantial among consumers as median contributions to total micronutrient intakes were up to 32%. This is in accordance with the WHO, stating widely distributed and -consumed fortified foods have the potential to improve nutritional status of large proportion of the population [3].

Unlike for vitamin A, the higher habitual vitamin D-intakes of fortified fat users did not result into a different evaluation on the risk on inadequate intakes. However, the total habitual intake distribution for the total user population increased, compared to non-users. In Finland similar results were found after the introduction of a new decree recommended to fortify liquid dairy products (0.5 µg/100 ml) and fat spreads (excluding butter; 10 µg/100 g) with vitamin D in 2002 [20]. Among the total population, vitamin D status improved, however among specific age groups the vitamin D status was still too low in 2004 and intakes of the total populations were below the recommendations in 2007 [20]. Even after a doubling of the mean intake of the population compared to 2002, after the National Nutrition Council of Finland doubled the recommendations of the decree in 2012, women remained below the recommended daily intake of 10 µg [20]. On the other hand, serum vitamin D concentrations were sufficient in 2011 and 2012 for both men and women [20, 21]. Research into the vitamin D-status of the Dutch users and non-users could give more insight into the true effects of the vitamin D fortification of fats and also the risks on deficiency in the Netherlands.

Vitamin D-intake from regular and fortified foods is not the only source of vitamin D. Vitamin D is also produced by the skin from sunlight, and if that is not sufficient, food supplements are advised by the Dutch government to specific subgroups. To prevent deficiency symptoms, the Health Council of the Netherlands recommends a daily vitamin D-supplement for specific population groups, including young children, women above 50 years old, elderly and everyone with a darker skin, or low sun exposure [22]. The total vitamin D-intake from foods and supplements of the Dutch population is below the AI with a median intake of 3.5 µg for the total population and between 4 and 5 µg for adults above 50 years old [9]. This is above the AI assuming adequate sun exposure, but below the AI assuming inadequate sun exposure. It is from the DNFCS not known which subjects have a darker skin or have insufficient sun exposure. It is important to also perform vitamin D status research in the general population, with specific analyses separated for users and non-users of supplements and perhaps users of fortified foods. In addition, insight in the commitment to the supplementation advice is needed and if low, the reasons behind this. This may help policy makers to improve the adherence to the supplementation advice, or to decide on other policy measures to increase the vitamin D intake, if necessary.

The dietary reference values EAR and AI established by the Health Council of the Netherlands and used in current study have different levels of evidence [12]. An EAR meets the need of 50% of the population and have a relatively strong evidence. With the EAR cut-point method it is possible to estimate the proportion with inadequate intakes, under some conditions [16]. When evidence is limited, an AI is established, which is a level of intake assumed to be adequate for the total population. This means the AI is probably higher than the individual requirement for a large part of the population, if the individual requirement would be known. The current study showed no statement could be made on the risk on inadequate vitamin D and E intakes for (most of the) adults and on the calcium intakes for the elderly in both non-users as users. Additional research on nutritional status or health effects is needed to confirm or contradict these possible inadequate intakes.

In the current study, we analyzed the proportion below the EAR, using the EAR cut-point method. This method is a short-cut of the Probability Approach (PA) [16]. Both the EAR cut-point method and the PA have several assumptions. For the PA these are: intakes and requirements are assumed to be independent, the mean and variance of the requirement distribution must be known and the form of the requirements distribution must be known or assumed. Also, for the EAR cut-point method the assumptions include intakes and requirements to be independent. Other assumptions for the EAR cut-point method include a symmetric distribution of requirements and a small variance of the distribution of requirements relative to the variance of the distribution of intakes. For all the nutrients we analyzed, there is a general assumption the assumptions of the EAR cut-point method are met [16]. However this is based on scarce data and mainly scenario studies. The assumed variance of requirement distribution varies between 10 and 25%, especially with high variance the difference with the variance of intake distribution may become less. It is recommended to study the differences in outcome between EAR cut-point method and probability approach in more depth using real-life data. In our study we also compared the intake distribution with the UL, by estimating the proportion with intakes above the UL. This is a common approach, however, Carriquiry and Camano-Garcia (2006) proposed to assess the percentage below the UL [23], because intakes below the UL are considered safe, however, intakes above the UL are not per definition unsafe. But, with intakes above the UL, there is a risk on adverse health effects, depending for example on amount, duration and sensitivity. At this moment no framework mirroring the DRI-paradigm is available, therefore the proportion with excessive intakes cannot be quantified. In our study intakes remained below the UL for most nutrients, indicating safe intakes.

The covenant between food producers and Dutch authorities appeared to be an effective strategy to increase micronutrient intakes. However it only reaches the consumers of these types of foods. In our study 16% of the population did not consume these fortified fats on at least one of the recall days. Changing the fortification policy could potentially increase the intake and reach a larger number of subjects. Besides increasing the intake and have more subjects fulfilling the requirements, safe intakes are also important to include. In the current Dutch fortification and supplementation policy, assuring safe intakes for the total population is important. As a result, for instance, the legal maximum levels for vitamin D addition to fortified fats, voluntary fortified other foods and dietary supplements are estimated in accordance. Therefore, the total intake would not exceed the UL. Consequently, a potential change in the fortification policy will affect the supplement legislation. In the Netherlands, strict regulations for food supplements are present for vitamin A, B6 and D. For all other vitamins, the vitamin content of supplements should not be harmful for public health [24].

The current study showed that users of margarines and other plant-based fats had more often a lower educational level, a lower level of urbanization and a Dutch ethnicity than non-users. There is evidence that dietary patterns can differ between population groups. In Belgium, eating meat or fish almost every day was associated with a lower educational level and a lower urbanization level, compared to those having a more plant-based diet [25]. Also, a higher educational level was associated with a higher level of fruits and vegetables consumption [26]. Dinnissen et al. (2021) showed also in the Netherlands a higher vegetable consumption and a lower meat consumption was observed among those with a higher education, compared to those with a lower, using data from the DNFCS 2007–2010 and 2012–2016 [27]. In our study we found higher habitual vitamin A-, B2- and B6-intakes among users of fortified fats, which are nutrients naturally present in animal based products. There is a possibility the higher intakes of these nutrients among users are, besides the intake from fortified fats, explained by a possible higher animal based diet of the users of fortified fats. On the other hand, vitamin B12 is only present in animal based products, but median intakes of users did not differ from non-users in our study. Therefore, the effect of possible different diet patterns across fortified fat users and non-users remains unclear and could be investigated further.

Van Rossum et al*.* showed that within the Dutch population, total intakes from foods and supplements for adults are low for calcium, vitamin B2, B6, C, and folate [9]. The current study showed fortified fats contributed to the intake of, among others, calcium, vitamin B2, B6 and folate equivalents, indicating the fortification of fats may contribute to increase the intake of these nutrients. Also, habitual intakes of users were higher for these nutrients, compared to non-users. Although the allowance of voluntary food fortification in the Netherlands was not intended to increase micronutrient intakes, and to lower risks on inadequate intakes, it still may have a beneficial effect on the proportion with intakes below the EAR. Although the current study showed no higher risk on excessive intakes among users of fortified fats, monitoring of the micronutrient intakes is required to prevent excessive intakes and to change policy on fortification and/or supplementation. De Jong et al*.* [paper in publication process, accepted by EJON], showed voluntary fortification of other foods also contributed to higher micronutrient intakes among users of these foods, compared to non-users and did not result into excessive intakes. In addition, it is generally known that a mandatory fortification of many food types has larger effect on the population-intake as more subjects are user of at least some of these food types [3]. On the other hand, increasing the number of foods which may be fortified have to result into a reduction of the level of fortification for each food in order to protect the population for excessive intakes. Scenario studies can predict the result of such increases.

Current study showed the habitual vitamin A (RAE) intakes were higher among users of fortified fats, except for girls 1–17 year old. However, when we calculated the mean daily intake of users and non-users in this age-sex group from the two recall days, we did observe a higher intake among users. The habitual intakes in our study were based on two independent recall days per person, and calculated with SPADE using the 1-part model, intended for food components consumed daily by (virtually) everyone in the population. For vitamin A, however, some extreme intakes were observed due to the consumption of liver (products), influencing the within- and between-person variation and shifting the habitual intake distribution. Mean habitual intakes of non-users girls were 14% higher compared to the mean of the daily intakes, while the habitual intakes were 14% lower, resulting into similar habitual intakes in this group. Also within the DNFCS 2012–2016 when no distinction between users and non-users were made, the habitual vitamin A-intakes were influenced by the few extreme intakes resulting into 11% lower habitual intakes compared to the daily mean (data not shown; [9]). These extreme intakes are however, important to include to assess the proportion above the UL. A multipart model for total intake from 2 or more food sources might be more applicable for vitamin A [14], however since there are only a few subjects with extreme vitamin A-intakes and two days of intakes, this is not feasible at this moment. Future research may focus on how to deal with extreme intakes in the calculation of habitual vitamin A-intakes.

With encouraged fortification of a specific food group, non-users of this specific food group do not benefit. In our study, consumption of fortified fats appeared to be associated with education, urbanization and ethnicity. Increasing fortified fat consumption among higher educated people, people living in more urbanized areas and people with a non-Dutch ethnicity could contribute to higher vitamin A and D intakes, and possibly also the intake of other nutrients. In the current study 16% of the Dutch population was classified as non-users, however, there is a possibility subjects were misclassified, as classification was based on only two recall days. Two recall days do not reflect the food habits of the population, as these may shift throughout the days. In the DNFCS 2012–2016, we did ask about food habits with an extra questionnaire, unfortunately, use of fortified fats was not included. Therefore, it was not possible to identify the true users and non-users. For future studies it is recommended to collect more information about the use of fortified fats, e.g. via an additional questionnaire.

Although it was difficult to determine users and non-users with certainty, DNFCS 2012–2016 was designed with great care. The extensive questionnaire and extensive food consumption information linked to the NEVO-database provides the current study precise data about the population characteristics and intakes. NEVO is a comprehensive database, consisting of most of the foods consumed within the Dutch population. The coverage of the micronutrients reported in the current study within this database is high, ranging from 87 to 99%. This high coverages means that for most of the products within DNFCS 2012–2016 the micronutrient content is known. If micronutrient content was not known, we assumed micronutrient content was equal to zero. Also, since it is not possible to make conclusions solely on the intake of two recall days, as intakes may vary from day to day, we used SPADE to correct for those within-person variances. This resulted into habitual intakes for the total population, making it possible to draw conclusions based on usual intakes, rather than daily. These habitual intakes were compared to dietary reference values.

## Conclusion

Fortified fats are often consumed in the Dutch population. The fats have a large contribution to micronutrient intake for the encouraged added vitamins A and D. Intake distributions of users of fortified fats are higher for the other nutrients voluntary added to fats, besides vitamin A and D. Therefore fortified fats may play a role in increasing micronutrient intakes. Increasing nutrient levels of fortified foods in general has to be done with care, as the risk on excessive intakes remains.

## Data Availability

Data of the DNFCS 2012–2016 are available on request from https://www.rivm.nl/en/dutch-national-food-consumption-survey/data-on-request (Accessed on 20 May 2021).
